# Approaches to Research Ethics in Health Research on YouTube: Systematic Review

**DOI:** 10.2196/43060

**Published:** 2023-10-04

**Authors:** Joshua P Tanner, Courtney Takats, Hannah Stuart Lathan, Amy Kwan, Rachel Wormer, Diana Romero, Heidi E Jones

**Affiliations:** 1 CUNY Graduate School of Public Health and Health Policy New York, NY United States; 2 CUNY Institute of Implementation Science in Population Health New York, NY United States

**Keywords:** data anonymization, research ethics, ethics, informed consent, public health, research, social media, YouTube

## Abstract

**Background:**

YouTube has become a popular source of health care information, reaching an estimated 81% of adults in 2021; approximately 35% of adults in the United States have used the internet to self-diagnose a condition. Public health researchers are therefore incorporating YouTube data into their research, but guidelines for best practices around research ethics using social media data, such as YouTube, are unclear.

**Objective:**

This study aims to describe approaches to research ethics for public health research implemented using YouTube data.

**Methods:**

We implemented a systematic review of articles found in PubMed, SocINDEX, Web of Science, and PsycINFO following PRISMA (Preferred Reporting Items for Systematic Reviews and Meta-Analyses) guidelines. To be eligible to be included, studies needed to be published in peer-reviewed journals in English between January 1, 2006, and October 31, 2019, and include analyses on publicly available YouTube data on health or public health topics; studies using primary data collection, such as using YouTube for study recruitment, interventions, or dissemination evaluations, were not included. We extracted data on the presence of user identifying information, institutional review board (IRB) review, and informed consent processes, as well as research topic and methodology.

**Results:**

This review includes 119 articles from 88 journals. The most common health and public health topics studied were in the categories of chronic diseases (44/119, 37%), mental health and substance use (26/119, 21.8%), and infectious diseases (20/119, 16.8%). The majority (82/119, 68.9%) of articles made no mention of ethical considerations or stated that the study did not meet the definition of human participant research (16/119, 13.4%). Of those that sought IRB review (15/119, 12.6%), 12 out of 15 (80%) were determined to not meet the definition of human participant research and were therefore exempt from IRB review, and 3 out of 15 (20%) received IRB approval. None of the 3 IRB-approved studies contained identifying information; one was explicitly told not to include identifying information by their ethics committee. Only 1 study sought informed consent from YouTube users. Of 119 articles, 33 (27.7%) contained identifying information about content creators or video commenters, one of which attempted to anonymize direct quotes by not including user information.

**Conclusions:**

Given the variation in practice, concrete guidelines on research ethics for social media research are needed, especially around anonymizing and seeking consent when using identifying information.

**Trial Registration:**

PROSPERO CRD42020148170; https://www.crd.york.ac.uk/prospero/display_record.php?RecordID=148170

## Introduction

Despite the addition of several social media platforms in the last 5 years, YouTube remains the second-most popular platform [1], with 81% of US adults reporting use in 2021 and 11.7 billion visits per month in 2023 [2]. YouTube is a diverse ecosystem with various contributors, including individual content creators, institutional and organizational accounts, and government accounts [3]. YouTube’s accessibility and ease of use make it a source of health information [4]. An estimated 35% of US residents use web-based sources to self-diagnose a condition [5,6]. YouTube can also be used as a data source, in terms of both content and public response to content (eg, likes and comments), and is being used increasingly by public health researchers to explore trends in viewership, popularity, engagement, and content accuracy [7].

Many researchers consider social media data as publicly available and thus not requiring ethical oversight. However, examples of breaches in research ethics using social media data have been identified. For example, a review of ethical breach case studies from 2019 described a study that disclosed an individual’s HIV status without the individual’s consent [8]. Further, videos posted do not always clearly indicate consent from individuals included in them. A 2020 study of YouTube videos depicting patients undergoing medical procedures found that none of the 41 videos identified indicated that the patient consented to the recording and posting of the video [9]. Finally, clarity is lacking among social media users on the extent to which the data they provide to social media platforms are public [10]. While numerous previous systematic reviews have explored the quality and accuracy of YouTube’s health-related content [4,11-14], previous reviews have not synthesized findings around approaches to research ethics, an area of increasing attention and concern [15]. We therefore implemented a systematic review of research from 2006 to 2019 investigating the use of YouTube for public health research in terms of approaches to research practices around ethics.

## Methods

### Search Strategy

The full search protocol for this review is registered under PROSPERO (CRD42020148170). This review follows PRISMA (Preferred Reporting Items for Systematic Reviews and Meta-Analyses) guidelines [16]. We searched PubMed, SocINDEX, Web of Science, and PsycINFO on November 6 and 7, 2019, using the search query ([“Social media” OR twitter OR tweet* OR facebook OR instagram OR youtube OR tumblr OR reddit OR “web 2.0” OR “public comments” OR hashtag*] and [“public health” OR “health research” OR “community health” OR “population health”]). We focused on YouTube only; findings for X (formerly Twitter) and Facebook are presented elsewhere [17,18].

### Eligibility Criteria

English-language peer-reviewed journal articles concerning health and YouTube published between January 1, 2006 (the first full year after YouTube’s founding), and October 31, 2019 (when we completed our search), were eligible. This review assessed research ethics practices when using YouTube as a data source; studies using YouTube as a source for an intervention, health information dissemination, or health education and promotion were excluded (Textbox 1).

**Eligibility criteria.**

**Inclusion criteria**

- Original research papers
- English
- YouTube only
- Public health– or health-based content
- Secondary analysis of publicly available data

**Exclusion criteria**

- Systematic review, literature review, opinion paper, and theoretical paper
- Non-English
- Facebook, Twitter, Instagram, other social media platforms, and multiple social media platforms (even if YouTube was included)
- Non–health-related content
- Analysis and evaluation of social media campaigns, interviews and surveys about social media, marketing or sales research, use of social media for study recruitment only, and studies testing use of social media as intervention

### Study Selection

After removing duplicates, 2 blinded reviewers screened each title and abstract or full text, if not clear from the title and abstract, for eligibility. We further screened the eligible studies’ reference lists.

### Data Extraction

We extracted data using a standardized extraction form including fields for publication year, public health topic, outcome type, whether institutional review board (IRB) approval was sought, IRB determination, whether informed consent was sought, and whether identifying information was included (defined as any information that could help identify a YouTube user, content creator, or commenter). We categorized health conditions as noncommunicable and chronic diseases, infectious diseases, mental health and substance use, injury, or other. We categorized outcome types as descriptive (content analysis yielding video themes through quantitative or narrative comparisons), quality (measuring video quality), attitudes (measuring the video’s/commenters’ sentiment), utility (measuring a video’s usefulness to viewers), or other (behavioral analysis, accessibility, etc). JPT performed the initial data extraction, and HEJ reviewed all data for accuracy. As this review focuses on ethical research processes, we did not include an assessment of quality or risk of bias.

### Analysis Presentation

We report the frequency and percentage of research ethics practices overall and by whether they included user-identifying information. We provide references in the main text for results with ≤10 studies to facilitate readability. Those with >10 studies in a result can be verified in Table S1 in Multimedia Appendix 1.

## Results

**Figure 1:**
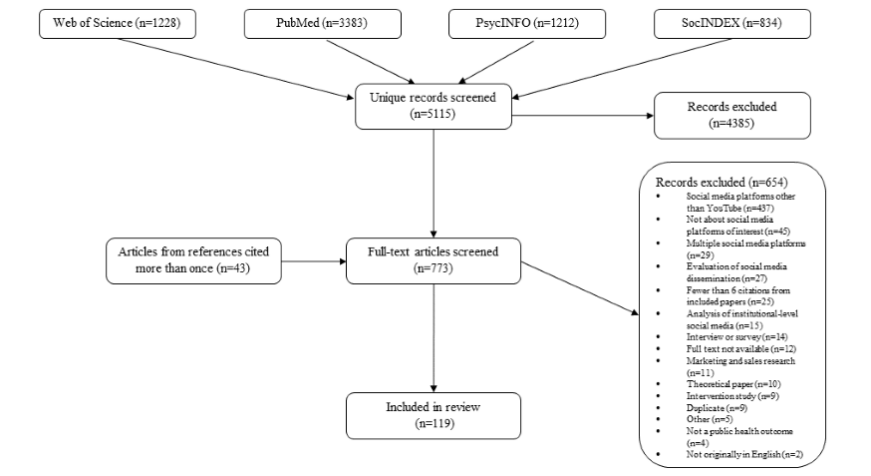
PRISMA (Preferred Reporting Items for Systematic Reviews and Meta-Analyses) flow chart of systematic review of public health studies of YouTube (2006-2019).

After removing duplicates, we reviewed 5115 unique studies, of which 119 met eligibility criteria (Figure 1) [19-137].

### Topic and Outcome Measures

The most common health topic was noncommunicable or chronic disease (44/119, 37%), followed by mental health and substance use (26/119, 21.8%) and infectious disease (20/119, 16.8%). Within chronic diseases, cancer was the most prevalent (10/119, 8.4%) [25,28,29,35,58-60,79,98,124], while vaccination was the most prevalent topic among articles on infectious disease (7/119, 5.9%) [21,34,52,57,62,85,136]. Most studies used content analysis to describe the videos’ themes (89/119, 74.8%), quality or utility (35/119, 29.4%), and public opinion or attitudes (31/119, 26.1%; Table S1 in Multimedia Appendix 1).

### Ethical Considerations

Most (82/119, 68.9%) articles did not mention ethical considerations in the study design or data collection, or the authors stated that their study did not meet the definition of human participant research (16/119, 13.4%). Of the 15 (12.6%) sent to the IRB for review, 12 (10.1%) were deemed to be nonhuman research by the IRB, and 3 (2.5%) [57,65,111] received IRB approval (Table 1). None of the 3 IRB-approved studies contained identifying information, one having been explicitly instructed not to include identifying information by their ethics committee [57].

**Table 1:** Frequency of research ethics practices by the presence of identifying information.

Research ethics practice			Total (N=119), n (%)		Included identifying information (n=33), n (%)		Did not include identifying information (n=86), n (%)
**Sought IRB^a^ review**							
	Yes, IRB deemed not human participants research	12 (10.1)		3 (9.1)		9 (10.5)	
	Yes, IRB approved	3 (2.5)		0 (0)		3 (3.5)	
	Not clear, authors deemed not human participants research	16 (13.4)		3 (9.1)		13 (15.1)	
	No	88 (73.9)		27 (81.8)		61 (70.9)	
**Sought informed consent**							
	Yes	1 (0.8)		1 (3)		0 (0)	
	No	118 (99.2)		32 (97)		86 (100)	

^a^IRB: institutional review board.

In total, 33 (27.7%) studies contained identifying information, which included video titles (18/119, 15.1%), direct quotations of comments or videos (14/119, 11.8%), URLs for videos (12/119, 10.1%), names of video content creators (7/119, 5.9%) [29,53,84,103,126,127,131], and video screen captures (2/119, 1.7%) [29,127]. One study that included direct quotes excluded usernames in an attempt to anonymize the data, stating they did so because the YouTube users were unable to consent to participation in the study [108]. Two additional studies chose not to include videos that depicted perceived adolescents or children [84,102].

One study on diabetes burnout in people with type 1 diabetes with personal stories from individuals with type 1 diabetes in the videos sought informed consent from content creators through email or by commenting on the video itself [19]. The study used an opt-out solicitation and received no requests to be removed from the study.

## Discussion

### Overview

We found some variation in research practice around ethical review for studies using YouTube data. Nearly three-quarters did not seek IRB review, considering YouTube publicly available data and thus not human participant research. Nevertheless, 12.6% (15/119) sought ethical review. Further, some researchers chose to anonymize their presentation of results or not to include identifiable content from children or adolescents; one study in Canada indicated that their ethical review board required content anonymization [57]. This variation in practice suggests ambiguity around privacy, personal identifiers, consent, and definitions of nonhuman participant research when using YouTube data.

This systematic review has several limitations. The review includes articles published from 2006 to 2019; as such, any changes to and evolution of the ethical and methodological use of social media data for research since 2019 are not captured. Additionally, given the breadth of public health research and the inclusion of only English-language articles, some studies may have been missed.

Given the continual debate about the definition and complexity of “public space” in social media and the shift away from a binary view of public versus private [138,139], and toward a consideration of intended use and “imagined audiences” [140], clearer guidelines are needed in social media research ethics. When studies include videos of real people sharing their personal health-related experiences, the notions of privacy and consent are blurred, with the possibility of social harm to nonconsented individuals.

### Conclusions

Clearer ethical guidelines around the use of “publicly available” social media data in scientific research are needed.

## Appendix

Multimedia Appendix 1 Individual listing of public health topic, whether study included identifying information, IRB processes of included articles (n=119) for systematic review of public health studies of YouTube 2006-2019.

Multimedia Appendix 2 PRISMA checklist.

## Abbreviations

IRB: institutional review board
PRISMA: Preferred Reporting Items for Systematic Reviews and Meta-Analyses
